# Early Salivary miRNA Expression in Extreme Low Gestational Age Newborns

**DOI:** 10.3390/life12040506

**Published:** 2022-03-30

**Authors:** Roopa Siddaiah, Lucy Emery, Heather Stephens, Ann Donnelly, Jennifer Erkinger, Kimberly Wisecup, Steven D. Hicks, Yuka Imamura Kawasawa, Christiana Oji-Mmuo, Shaili Amatya, Patricia Silveyra

**Affiliations:** 1 Department of Pediatrics, Penn State Health Children’s Hospital, Hershey, PA 17036, USA; hstephens@pennstatehealth.psu.edu (H.S.); kwisecup@pennstatehealth.psu.edu (K.W.); shicks1@pennstatehealth.psu.edu (S.D.H.); cjimmuo@pennstatehealth.psu.edu (C.O.-M.); samatya@pennstatehealth.psu.edu (S.A.); 2 Penn State Health College of Medicine, Hershey, PA 17036, USA; lemery@pennstatehealth.psu.edu; 3 Department of Respiratory Therapy Penn State Health Children’s Hospital, Hershey, PA 17036, USA; adonnelly@pennstatehealth.psu.edu (A.D.); jerkinger@pennstatehealth.psu.edu (J.E.); 4 Departments of Pharmacology, Biochemistry and Molecular Biology, Penn State Health College of Medicine, Hershey, PA 17036, USA; yimamura@pennstatehealth.psu.edu; 5 Department of Environmental and Occupational Health, School of Public Health, Indiana University, Bloomington, IN 47405, USA; psilveyr@iu.edu

**Keywords:** miRNA, BPD, tracheal aspirate, saliva sample, chronic lung disease

## Abstract

Background: MicroRNAs (miRNA) are small non-coding RNAs that regulate gene expression playing a key role in organogenesis. MiRNAs are studied in tracheal aspirates (TA) of preterm infants. However; this is difficult to obtain in infants who are not intubated. This study examines early salivary miRNA expression as non-invasive early biomarkers in extremely low gestational age newborns (ELGANs). Methods: Saliva was collected using DNA-genotek swabs, miRNAs were analyzed using RNA seq and RT PCR arrays. Salivary miRNA expression was compared to TA using RNA seq at 3 days of age, and longitudinal changes at 28 days of age were analyzed using RT PCR arrays in ELGANs. Results: Approximately 822 ng of RNA was extracted from saliva of 7 ELGANs; Of the 757 miRNAs isolated, 161 miRNAs had significant correlation in saliva and TA at 3 days of age (r = 0.97). Longitudinal miRNA analysis showed 29 miRNAs downregulated and 394 miRNAs upregulated at 28 days compared to 3 days of age (adjusted *p* < 0.1). Bioinformatic analysis (Ingenuity Pathway Analysis) of differentially expressed miRNAs identified organismal injury and abnormalities and cellular development as the top physiological system development and cellular function. Conclusion: Salivary miRNA expression are source for early biomarkers of underlying pathophysiology in ELGANs.

## 1. Introduction

MicroRNAs (MiRNAs) are small, highly conserved, non-coding RNAs which have complex roles in a variety of biochemical processes [1]. Although MiRNAs are non-coding they are known to regulate protein production through regulation of mRNA translation. MiRNAs act as negative regulators of gene expression by inhibiting the translation or promoting the degradation of target mRNAs. Additionally, an altered expression of a single miRNA can influence an entire gene network because a single miRNA can exert effects in multiple target genes [1]. It is therefore possible that dysregulation in miRNA profiles is responsible for various medical conditions. Therefore, studying MiRNAs may provide insight into the regulatory mechanisms and potential therapeutic targets for the treatment of disease.

Exosomes and exosomal miRNAs may also regulate cell differentiation and tissue and organ development [2]. Exosomes are lipid wrapped vesicles that transport proteins and genetic material between cells. Exosomes merge with and then release their contents into the receiving cell. This gives them an extremely important function in cell-to-cell communication [3]. Exosomes contain a wide variety of proteins, nucleic acids, and lipids, and they are present in many, if not all, bodily fluids including blood, saliva, urine, and cerebrospinal fluid. One of the contents of exosomes are miRNAs. These cell-secreted miRNAs are very stable and can be taken up by cells in the surrounding tissue, or if the vesicles enter circulation, they can reach more distant sites [1]. Recent literature has demonstrated that the molecular constituents of exosomes, especially exosomal proteins and miRNAs, have the potential to become novel biomarkers for clinical diagnosis.

Recent improvements in neonatal care have resulted in increased survival rates of very premature infants. As a result of increased survival rates, there is accompanied corresponding increase in the complications associated with preterm birth. Premature infants are at high risk for developing bronchopulmonary dysplasia (BPD), a condition characterized by chronic inflammation and inhibition of lung development leading to incompetent gas exchange [4]. The pathogenesis of BPD is multifaceted involving environmental factors such as hyperoxia, ventilation-induced lung injury, and inflammatory responses [4]. Genetic influences also play a role affecting gene regulatory pathways involved in alveolar and vascular development [5]. Recent studies have suggested that circulating miRNAs, exosomal microRNAs, and exosomes may play a crucial role in this genetic regulation [2,6]. Hyperoxia related stress injury disrupts vascularization and alveolarization via miRNAs during lung development, contributing to the development of BPD and associated comorbidities [6].

Past studies assessing the roles of miRNAs in BPD have analyzed their content in tracheal aspirates (TAs) of newborns and infants [2,6,7,8]. Studies have found that TAs of extremely premature infants contain miRNA signatures associated with severe BPD 8. These may serve as potential biomarkers for disease severity in infants with BPD [7,8]. Additionally, specific mRNA-miRNA signatures in TAs may serve as indicators of mechanisms of BPD pathogenesis, a consequence of extreme prematurity 8. Despite strong evidence of the role of miRNAs in BPD development, there has not been research on salivary BPD miRNAs in the past, although recent studies using new technologies have validated a wide range of salivary biomarkers for other conditions and disease processes [9,10]. In fact, there have been so many advances in analytical techniques that a whole area of research coined “salivaomics” has been established. Salivaomics investigates the salivary proteome, transcriptome, miRNAs, metabolome, and microbiome [11,12]. Human saliva harbors coding and noncoding RNAs, including over 1000 miRNAs and more than 3000 species of mRNAs [10,11]. Past studies have focused on saliva biomarkers in association with oral disease [9,10]. However, newer research has shown that salivary biomarkers not only arise in correlation with oral diseases, but also disorders of distal tissues and organs, potentially including the lungs [11]. This suggests that saliva may represent information capable of communicating the presence of disease throughout the body [9,12].

Maron et al. has demonstrated saliva as a means for miRNA biomarker discovery could revolutionize care because its collection is fast, easy, inexpensive, and non-invasive [13,14]. Additionally, it would allow us to analyze miRNA and exosomal biomarkers in infants who are not intubated. The hypothesis of our study is that salivary samples can be easily obtained in ELGANs and miRNAs can be analyzed as early as within the first 3 days of life, and also can be tracked for longitudinal dynamic changes over time. As a secondary aim, we assessed the overlapping expression of the salivary miRNAs with simultaneously collected TAs from the same infant at the same timeframe. Using this information, we conducted bioinformatic analyses to identify putative target pathways associated with prematurity, BPD, and its associated sequelae.

## 2. Materials and Methods

We conducted a prospective observational study on extremely low gestational age newborns (ELGANs) (born at less than 28 weeks of gestation) admitted to neonatal intensive care unit at Penn State Health Children’s Hospital. This study was reviewed by the institutional review board and approved under study # STUDY00000482 for patient enrollment. We included infants born less than 28 weeks of gestation with no underlying chromosomal abnormalities or major congenital malformations such as cyanotic cardiac defects, omphalocele, Pierre Robin sequence, etc. Once subjects were identified, informed consent was obtained from the parents or legal guardians. We collected salivary samples from infants at 3 and 28 days of age using DNA genotek saliva collection swabs as described in previous studies [15,16]. The swab from the kit was placed in the oral cavity of the infants for 60 s. To prevent agitation or local irritation to the delicate mucus membrane the swab was placed in the mouth without any rubbing or movement for the 60 s. This method of sample collection was well tolerated even in infants including those who needed high frequency jet ventilation. There was no variation in heart rate, or oxygen saturation during the sample collection which would indicate a stress response to sample collection. We also simultaneously collected TAs in infants who were intubated at birth for mechanical ventilation during their routine care at 3 days of age as described before [8]. Collected samples were immediately stored at −80 °C until ready to be analyzed.

Initial sample analysis was conducted on saliva samples collected at 3 days of age using RNA seq. RNA was extracted using Quick-RNA Microprep purification kits (Zymo Research). RNA quality and quantity were determined by RNA Pico BioAnalyzer (Agilent technologies). RNA showed typical size distribution for the small RNA and quantities ranged between 33.90 and 922.50 ng for saliva and 13.14 and 283.50 ng for TA exosome samples, respectively. For RNA seq analysis, small RNA libraries were prepared from 5–250 ng total RNA using the QIAseq miRNA Library Kit (QIAGEN) as per the manufacturer’s instructions. This system offers a built-in Unique Molecular Identifier (UMI) application which we used to eliminate possible PCR duplicates in sequencing datasets and therefore facilitate unbiased gene expression profiling. The unique barcode sequences were then incorporated in the adaptors for multiplexed high-throughput sequencing. The final product was assessed for its size distribution and concentration using a BioAnalyzer High Sensitivity DNA Kit (Agilent Technologies). All RNA seq experiments were conducted at the Penn State College of Medicine Genome Sciences Core Facility.

The libraries were pooled and diluted to 3 nM using 10 mM Tris-HCl, pH 8.5 and denatured. The denatured libraries were then loaded onto an SP flow cell on an Illumina^®^ NovaSeq 6000 instrument and run for 68 cycles according to the manufacturer’s instructions. De-multiplexed sequencing reads were generated using Illumina bcl2 fastq (released version 2.20.0.422), allowing no mismatches in the index read. The resulting multiplex high-throughput sequencing data was mapped, and UMI analysis was conducted via the GeneGlobe Data Analysis Center (QIAGEN) [17]. The detailed alignment workflow is available from QIAGEN (Ref.: https://ngsdataanalysis2.qiagen.com/handbooks/HB-2608-001_SP_Qseq_miRNA_Quantification_1118_WW_20181106_BA_12072018.pdf (accessed on 12 July 2021)) Similar technique was used to analyze TA samples after isolating exosomal RNA using the ExoQuick reagent (System Biosciences) and total RNAs with the Quick-RNA Microprep purification kit (Zymo Research). Alignment rate was 92% +/− 7% across all the the samples. We were unable to extract salivary exosomes from samples that were collected and stored using DNA genotek saliva collection kits. We believe the reason for not being able to extract the exosomes was due to the lytic nature of ORA-100 chemistry that disrupted the exosomes while still preserving its RNA content. The DESeq2 R package [18] was used to identify miRNA features with similar expression patterns between paired TA and saliva samples. Similarly expressed miRNAs were identified as those with adjusted *p*-value > 0.1 (calculated by the Benjamini-Hochberg method) [19] when comparing miRNAs from saliva vs. TA and absolute fold change <2. The list of similarly expressed small RNAs was subjected to Pearson’s correlation analysis.

To validate different techniques in miRNA isolation and quantification in ELGANs, we also analyzed the saliva samples collected at 3 and 28 days of age using RT PCR arrays. For this, RNA was extracted from saliva samples using the Norgen miRNA purification kit (Norgen Biotek Corp, Thorold, ON, Canada). The expression of 754 unique miRNAs was measured using TaqMan^®^ Array Human MicroRNA Cards (Human A + B Card Set v3, Thermo Fisher, Waltham, MA, USA) and Megaplex™ RT Primers (Thermo Fisher) in a QuantStudio 12 K Flex Real-Time PCR system, following the manufacturer’s protocol [20]. Cycle threshold (Ct) values were extracted and analyzed in the manufacturer’s online analysis software (Thermos Fisher cloud) using the Relative Quantification application module [21]. We processed the data by applying global mean normalization [22], setting mean Ct values < 32. We conducted statistical analyses for time point comparison of salivary miRNAs expression using *t*-tests, and we calculated adjusted *p*-values < 0.1 via Benjamini-Hochberg method to control the false discovery rate (FDR). All RT PCR array card experiments were conducted at University of North Carolina at Chapel Hill Biobehavioral Laboratory Core Facility.

## 3. Results

### 3.1. Patient Demographics

We enrolled 7 infants in our study. We obtained salivary samples at 3 days and at 28 days of age. We also obtained TAs by 3 days of age. The infant characteristics are shown in Table 1. The average gestational age was 25 4/9 weeks (ranging from 23 5/7 to 271/7) with an average birth weight of 617 g (ranging from 380–1010 g). All infants were intubated at birth hence we could obtain TAs within the first 3 days of age

### 3.2. Salivary and Tracheal Aspirate miRNA Expression at 3 Days of Age

**Figure 1:**
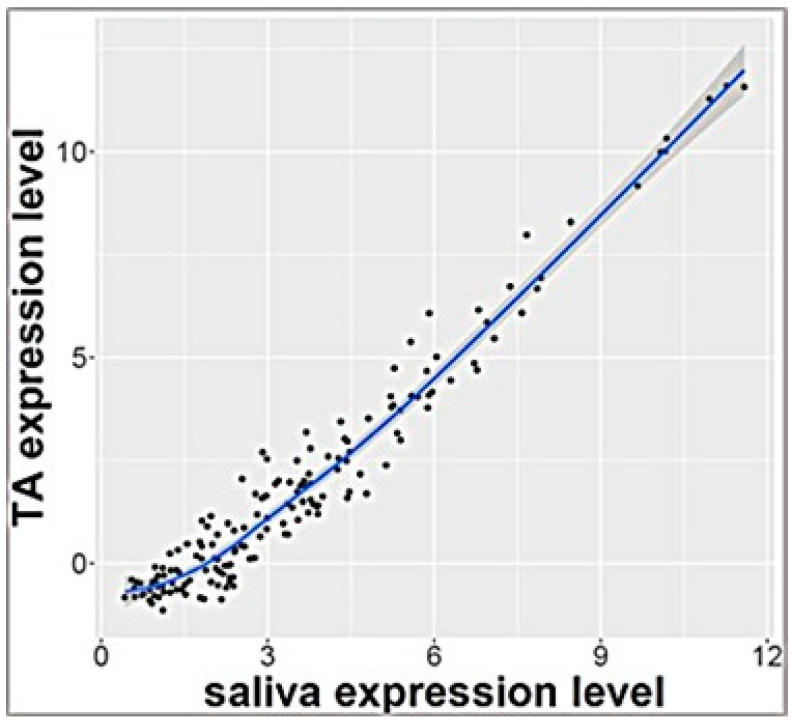
Correlation plot of 161 miRNAs with similar levels of expression in tracheal aspirate and saliva (r = 0.97).

RNA sequencing analysis of salivary samples at 3 days of age from 7 ELGANs detected 757 miRNAs that were expressed in all samples. These miRNAs were also detected in exosomal miRNA profile from TAs that were collected simultaneously (*p* > 0.1 calculated by Benjamini Hochberg method) and <2 fold change expression between biofluids. Pearson’s analysis showed significant correlation of these 161 miRNAs (Supplemental Table S1) (r = 0.97) as shown in Figure 1. Top 10 miRNAs that were similarly expressed in both saliva and TAs are shown in Table 2

### 3.3. Longitudinal, Dynamic Salivary miRNA Expression at 3 and 28 Days of Age

**Figure 2:**
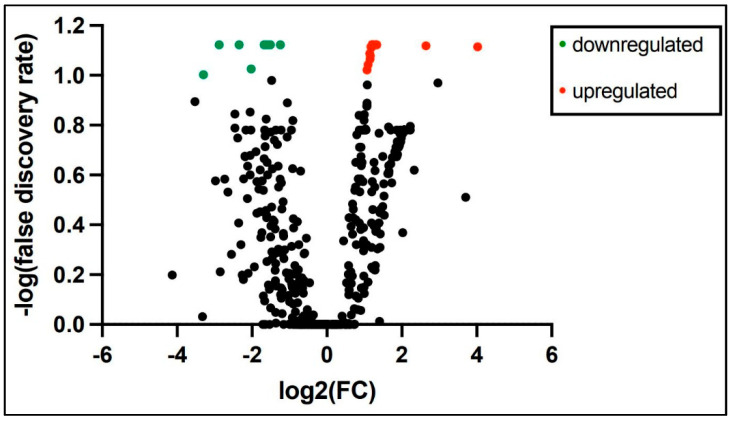
At least 2-fold change log expression of differentially expressed salivary miRNAs from 3 and 28 days of age in extremely premature born infants (green dot = downregulated and red dot = upregulated).

PCR array miRNA analysis performed at 3 and 28 days of age from 6 ELGANs detected expression of 758 miRNAs consistently in both time points. Analysis of miRNAs with at least 2-fold change log expression between the time points revealed 9 miRNAs to be down regulated and 219 miRNAs upregulated at 28 days when compared to 3 days of age (Supplemental Table S2) a volcano plot is shown in Figure 2. Top 10 differentially expressed miRNAs from 3 days of age to 28 days of age are shown in Table 3. Pathway analysis of similarly expressed miRNAs in saliva and tracheal aspirate at 3 days of age IPA analysis of similarly expressed miRNAs in both saliva and TA samples collected simultaneously at 3 days of age revealed molecular and cellular function and physiological system development and function pathways as the top associated networks. The top molecular and cellular functions included cellular movement, cellular response to therapeutics and cellular development. The top physiological system development and function pathways included organismal development, digestive system development and function and hepatic system development and function (Table 4). The molecular network showed top 5 predicted target genes AGO3, ALOX5, AGO2, ATM and CDKN2 A (Supplemental Table S3)

### 3.4. Pathway Analysis of Differentially Expressed miRNAs in Saliva at 3 and 28 Days of Age

Analysis of differentially expressed miRNAs in both saliva and tracheal aspirate samples collected simultaneously at 3 days of age revealed top molecular and cellular function, physiological system development and function pathways and top associated network functions. The top molecular and cellular functions included cellular movement, cellular response to therapeutics and cellular development. The top physiological system development and function included organismal development, digestive system development and function and hepatic system development and function (Table 5). The molecular network showed top 5 predicted target genes Akt, CG, Growth Hormone, Insulin and ERCC8 (Supplemental Table S4)

## 4. Discussion

**Figure 3:**
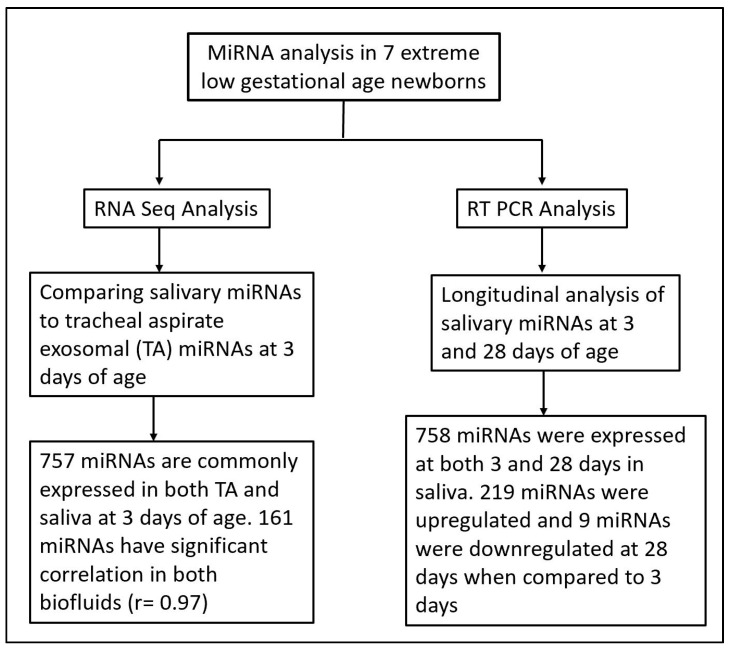
Flow chart illustrating miRNA analysis techniques and time points in saliva and tracheal aspirate samples from extreme low gestational age newborns.

There is increasing evidence of the role of miRNAs in cellular and organ development 21, and as potential biomarkers for disease severity and progression [22,23,24,25]. Despite strong evidence of the role of miRNAs in neonatal respiratory diseases such as BPD, no studies using salivary miRNAs as potential biomarkers and predictors of the disease pathogenesis have been conducted. In previous reports, we have successfully identified differentially expressed miRNAs in TAs of prematurely born vs. term infants, as well as infants with mild vs. severe BPD [7,8]. However, a consistent limitation of these studies was our inability to assess disease progression after extubation. In the present study, we overcame this challenge by not only assessing salivary miRNAs as a non-invasive, easily available sample, but we also conducted validation studies of saliva as a surrogate for TAs (Figure 3).

The current study identified multiple miRNAs that were similarly highly expressed in both saliva and TA samples at 3 days of age, which we believe represent systemic expression (given their high expression in more than one biofluid). Of particular interest is the highly expressed miR-204-5p, which has been previously isolated from exosomes and revealed to not only contribute to signaling pathways of cell differentiation, but also shown to have therapeutic potential in cancer cells [26,27]. This miRNA has also been studied as a key player in developmental lymphangiogenesis via transcriptional factors [28]. Similarly, miRNAs miR-21-3p and miR-28-5p are highly expressed in exosomes [29,30,31,32] and play a key role in cell proliferation and migration [33,33]. While we were unable to isolate exosomes from saliva with our collection method, comparing to an exosomal miRNA profile from TA has helped us identify the key miRNAs that are transported in exosomes and potentially represent a systemic miRNA expression in the salivary samples in ELGANs. Moreover, the top miRNA predicted pathways from both sample types highlighted developmental processes in the ELGANs and reflect ongoing cellular development and growth that are key for organismal development and maturation.

The current analysis also identified differentially expressed miRNAs between sample types and among timepoints. Analysis of the top miRNAs differentially expressed longitudinally showed miR-548a to have the largest log2 fold change. This miRNA has been shown to modulate cellular proliferation in cancer pathology [34,35] along with miR-199a [36], miR01224-3p [37] and miR-1228 [38].

Our study has several limitations, the most important being its small sample size. In addition, while ideally we would have preferred to isolate exosomes from saliva for miRNA analysis, we were unable to isolate them using our collection method. However, the strength of our study lays in the innovation of analyzing a readily available, non-invasive biofluid in ELGANs through a safe non-stressful collection method. This is attractive for patient recruitment especially in very small vulnerable population such as the neonatal population. Our results show that we were able to isolate miRNAs using two different techniques and establish longitudinal dynamic changes in the miRNA expression. This is of great value for longitudinal studies to evaluate ongoing-evolving process complications of prematurity. Thus, future studies with a larger sample size and assessing different BPD phenotypes are warranted and will help in the development of more readily available biomarker tools for pediatric disease diagnosis and progression.

## 5. Conclusions

With the growing population of extreme preterm infants, their neonatal lung disease morbidity and associated complications are more prevalent. Non-invasive biomarkers such as miRNAs that regulate growth and maturation of organs are valuable tools. Although representing a small sample, our study identified over 700 miRNAs using different techniques from saliva of ELGANs as early as 3 days of age. We have also identified longitudinal dynamic changes of miRNAs that are predicted to regulate cell movement, cellular response to therapeutics and cellular development using bioinformatic tools. These miRNAs and associated pathways represent potential targets of interest in studying various complications preterm birth.

## Figures and Tables

**Table 1:** Patient demographics (*n* = 7). AGA: appropriate for gestational age, SGA: small for gestational age.

Patient Demographics	
Gestational Age	25 4/7 (23 5/7–28 3/7)
Birth weight in grams	617 (38–1010)
Gender	M:F: 3:4
Race: *n*	White: 3 Other: 3 Asian: 1
AGA: SGA	3:4
Mode of delivery C-section: Vaginal birth	6:1

**Table 2:** Top 10 similarly expressed miRNAs in high intensity in salivary and tracheal aspirate samples at 3 days of age with significant FDR (adjusted *p* of <0.1).

miRNAs	Fold Change	Adjusted *p* Value	Expression Intensity
has-miR-204	0.885151	0.080909	9254.185
hashsa-miR-21-3p	−1.22323	0.063979	6147.197
hsa-miR-28-5p	−0.67488	0.027228	1980.699
hsa-miR-218-2-3p	−0.46345	0.055106	1350.412
hsa-miR-3 has5p	−1.03647	0.003002	1326.227
hsa-mihas0 e-5p	−1.52103	0.013793	1310.715
hsa-has-3679-5p	−1.01302	0.001325	1219.705
has-miR-363-3p	0.840106	0.001739	1219.248
hsa-miR-3153	0.52835	0.010092	1030.863
hsa-miR-449 b-5p	−1.15254	0.000161	974.736

**Table 3:** Top 10 differentially expressed miRNAs with at least 2 log fold change from 3 days to 28 days of age in ELGANs.

miRNAs	Fold Change	Adjusted *p* Value	Up Ohasown Regulated
hsa-miR-548a	16.236	0.076833	Up-regulated
hsa-miR-199a	6.245	0.076064	Up-regulated
hsa-miR-1224-3P	2.513	0.07549	Up-regulated
hsa-miR-1288	2.482	0.07549	Up-regulated
hsa-miR-337-3p	2.474	0.07549	Up-regulated
hsa-miR-182	2.437	0.07549	Up-regulated
hsa-miR-554	2.347	0.07549	Up-regulated
hsa-let-7c	2.333	0.07549	Up-regulated
hsa-let-7a	2.32	0.07549	Up-regulated
hsa-let-7b	2.32	0.07549	Up-regulated
hsa-let-7e	2.32	0.07549	Up-regulated

**Table 4:** IPA analysis of miRNA expression similarly expressed in saliva and tracheal aspirate of extremely premature born infants at 3 days of age.

**Top Molecular and Cellular Functions**	***p* Value Range**
Cellular Development	4.83 × 10^−2^–2.39 × 10^−8^
Cellular Movement	3.77 × 10^−2^–2.74 × 10^−7^
Cellular Growth and Proliferation	4.83 × 10^−2^–9.35 × 10^−7^
Cell Cycle	4.83 × 10^−2^–1.24 × 10^−5^
Cell Death and Survival	4.72 × 10^−2^–1.06 × 10^−4^
**Top Physiological System Development and Function**	***p* Value Range**
Organismal development	4.58 × 10^−2^–3.96 × 10^−6^
Digestive System Development and Function	2.17 × 10^−4^–2.17 × 10^−4^
Hepatic System Development and Function	2.17 × 10^−4^–2.17 × 10^−4^
Organ Development	2.73 × 10^−2^–2.17 × 10^−4^
Embryonic Development	1.96 × 10^−2^–8.60 × 10^−4^

**Table 5:** Ingenuity Pathway Analysis to identify target pathways of differential longitudinal miRNA expression in saliva at 3 and 28 days of age (>log2 fold change).

**Top Molecular and Cellular Functions**	***p* Value Range**
Cellular Movement	3.55 × 10^−2^–5.78 × 10^−11^
Cellular Development	4.90 × 10^−2^–7.99 × 10^−11^
Cellular growth and Proliferation	4.92 × 10^−2^–7.99 × 10^−11^
Cell Death and Survival	4.92 × 10^−2^–5.06 × 10^−7^
**Top Physiological System Development and Function**	***p* Value Range**
Embryonic Development	4.19 × 10^−2^–1.97 × 10^−6^
Organismal Development	4.50 × 10^−2^–2.38 × 10^−6^
Digestive System Development and Function	4.79 × 10^−6^–4.79 × 10^−6^
Hepatic System Development and Function	4.79 × 10^−6^–4.79 × 10^−6^
Organ Development	4.19 × 10^−2^–4.79 × 10^−6^

## Data Availability

All the data will be uploaded to GEO online database and will be accessible upon request by the reviewers.

## Supplementary Materials

The following supporting information can be downloaded at: https://www.mdpi.com/article/10.3390/life12040506/s1, Table S1: List of 161 miRNAs with significant correlation by Pearson’s analysis in miRNA expressed in both TAs and saliva samples at Day 3 of life; Table S2: List of miRNAs with at least 2 fold change from Day 3 to Day 28 of age in saliva samples; Table S3: List of Molecular network with top 5 predicted target genes from IPA analysis of miRNAs commonly expressed in TAs and saliva at day 3 of age; Table S4: List of Molecular network with top 5 predicted target gene from IPA analysis of differentially expressed miRNA from Day 3 to Day 28 days of age in saliva samples.
